# Swelling Inhibition of Liquid Crystalline Colloidal Montmorillonite and Beidellite Clays by DNA

**DOI:** 10.1038/s41598-018-22386-7

**Published:** 2018-03-12

**Authors:** Naoya Yamaguchi, Shinya Anraku, Erwan Paineau, Cyrus R. Safinya, Patrick Davidson, Laurent J. Michot, Nobuyoshi Miyamoto

**Affiliations:** 1 Fukuoka Institute of Technology, Department of Life, Environment and Materials Science, 3-30-1 Wajirohigashi, Higashiku, Fukuoka 811-0295 Japan; 2 Laboratoire de Physique des Solides, CNRS, Univ. Paris-Sud, Université Paris-Saclay, 91405 Orsay Cedex, France; 3 Physics Department, Materials Department, and Molecular, Cellular and Developmental Biology Department, University of California, Santa Barbara, California 93106 United States; 4 Laboratoire Phenix, CNRS−Sorbonne Université−UPMC, UMR 8234, 4, Place Jussieu, 75252 Paris Cedex 5, France

## Abstract

Exploring the interaction of nucleic acids with clay minerals is important to understand such issues as the persistence in soils of biomolecules and the appearance of genetic polymers in prebiotic environments. Colloidal dispersions of double stranded DNA and clay nanosheets may also provide interesting model systems to study the statistical physics of mixtures of semi-flexible rods and plates. Here, we show that adding very small amounts of DNA to liquid-crystalline montmorillonite and beidellite smectite clay suspensions strongly widens the isotropic/nematic phase coexistence region. Moreover, a spectroscopic study shows that, upon DNA addition, the first DNA molecules adsorb onto the clay particles. Remarkably, synchrotron small-angle X-ray scattering experiments reveal that the average distance between the clay sheets, in the nematic phase at coexistence, decreases with increasing DNA concentration and that the inhibition of swelling by DNA becomes almost independent of clay concentration. We interpret this DNA-mediated attraction between clay nanosheets by bridging conformations of DNA strands (plates on a string structure). In addition to bridging, DNA chains can form “loops” between sections adsorbed on the same particle, giving rise to sheet repulsions due to protruding loops. This interpretation agrees with the observed inter-clay spacings being dependent only on the DNA concentration.

## Introduction

Clay minerals are major constituents of soils and control, to a large extent, reactivity in these natural environments. In particular, their interactions with biomolecules such as proteins and nucleic acids are of prime importance for various reasons. Firstly, nucleic acids and proteins being bio-produced macromolecules, their presence in soils is generally due to either release by excretion from microorganisms, plants, and animals, or lysis of dying cells^1,2^. Upon interaction with clay minerals, these molecules are protected from degradation, whether enzymatic or mediated by ultraviolet (UV) radiation^3–6^. They can then persist for long times in hostile environments and maintain their biological activity, which may also be significant in terms of prebiotic environments^7–9^. Furthermore, the binding of these biomolecules by clay minerals influences soil microorganisms, and reduces their availability as a source of carbon and/or nitrogen for microbes^9–11^. Secondly, the interaction between clay minerals and nucleic acids and/or nucleic acid building blocks may have played a crucial role in the origin of life. The hypothesis that mineral surfaces mediated the prebiotic formation of genetic polymers was first proposed by Bernal^12^ as early as 1951 and very numerous studies have been devoted to this topic, since then (refs^9,13,14^ and references within). In that context, particular attention has been devoted to clay minerals. Indeed, the Hadean seafloor (between 4.6 to 4.0 billion years ago) was mainly formed with mafic and ultramafic rocks, such as basalt, komatiite and peridotite, the alteration products of which are rich in Fe-Mg phyllosilicates (i.e. sheet silicates), either swelling or non-swelling^14,15^.

Whereas numerous studies have explored clay-nucleic acids interactions from the point of view of adsorption and surface reactivity, much fewer studies have considered this issue in terms of the mixing of two colloidal entities and the resulting new phase behaviour. In the field of statistical physics, colloidal mixtures of nanoparticles are presently the focus of much research activity^16–19^ since single-component colloidal dispersions are rather well understood^20,21^. The difference in size of two colloidal components gives rise to the attractive depletion interaction which often plays a major role in the destabilization of colloidal mixtures^22^. Moreover, mixtures involving at least one anisotropic (rod-like or disk-like) constituent have a rich phase diagram displaying liquid-crystalline phases. In this context, the (clay, DNA) systems considered here may illustrate the theoretical cases of mixtures of disks (clay) with either semi-flexible rods (short DNA) or Gaussian coils (long DNA). To date, both of these cases have been little explored theoretically and experimentally.

Two-component colloidal suspensions of hard rods and plates of about the same size have been explored to search for the elusive biaxial nematic phase^23^. For example, colloidal dispersions of boehmite nanorods and gibbsite nanoplates of roughly the same size were investigated^24,25^. Although no biaxial nematic phase appeared in this system, the phase diagram shows up to four distinct liquid-crystalline phases, including several different rod-rich and disk-rich nematic phases. Other examples are mixtures of sepiolite nanorods a few microns long with montmorillonite nanosheets about one micron in diameter^26^. This system has an unusual phase diagram with regions where an isotropic phase coexists with two nematic phases differing by their compositions.

Examples of two-component colloidal suspensions of spheres and plates are also scarce. For example, mixing gibbsite platelets of 95 nm diameter and 10 nm thickness with silica spheres 17 nm in diameter led to a strong widening of the isotropic/columnar phase coexistence region^27^. Mixtures of more anisotropic gibbsite platelets (190 nm diameter and 4 nm thickness) and small silica spheres of diameter either 30 or 74 nm have also recently been considered^28^. Their phase diagram showed a widening of the isotropic/nematic (I/N) biphasic region compared to the pure suspensions of gibbsite platelets. At high densities, an isotropic phase almost pure in spheres coexists with a nematic phase almost pure in disks. Moreover, the phase diagrams also showed regions where two distinct isotropic phases coexist with a nematic phase of intermediate density, which is quite uncommon. Somewhat similar observations were recently made with a system that consists in α-ZrP nanoplates of 700 nm diameter and silica spheres of 160 nm diameter. In addition, the different demixtion pathways of triphasic systems were determined^29^. Moreover, mixtures of beidellite clay nanosheets of 200 nm diameter and 0.7 nm thickness and small silica spheres of 22 nm diameter displayed some stabilization of the nematic phase with respect to the isotropic one, together with some weakening of the viscoelastic properties^30^.

Before describing our findings on the behaviour of the (clay, DNA) colloidal mixtures, we need to recall previous results regarding the single-component suspensions. Upon increasing concentration, in absence of DNA, aqueous suspensions of beidellite clay first display an isotropic phase, then an isotropic/nematic phase transition (with phase coexistence), a fluid nematic phase, and finally a sol/gel transition towards a nematic gel state^31^. The montmorillonite clay also shows mostly a similar behavior^32^. In contrast, aqueous DNA solutions, in absence of clay, only form isotropic phases in the range of concentration explored in this study (between ≈0.2 g/L and ≈4 g/L). Indeed, liquid-crystalline phases of DNA only appear at concentrations beyond ~100 g/L^33^.

**Figure 1:**
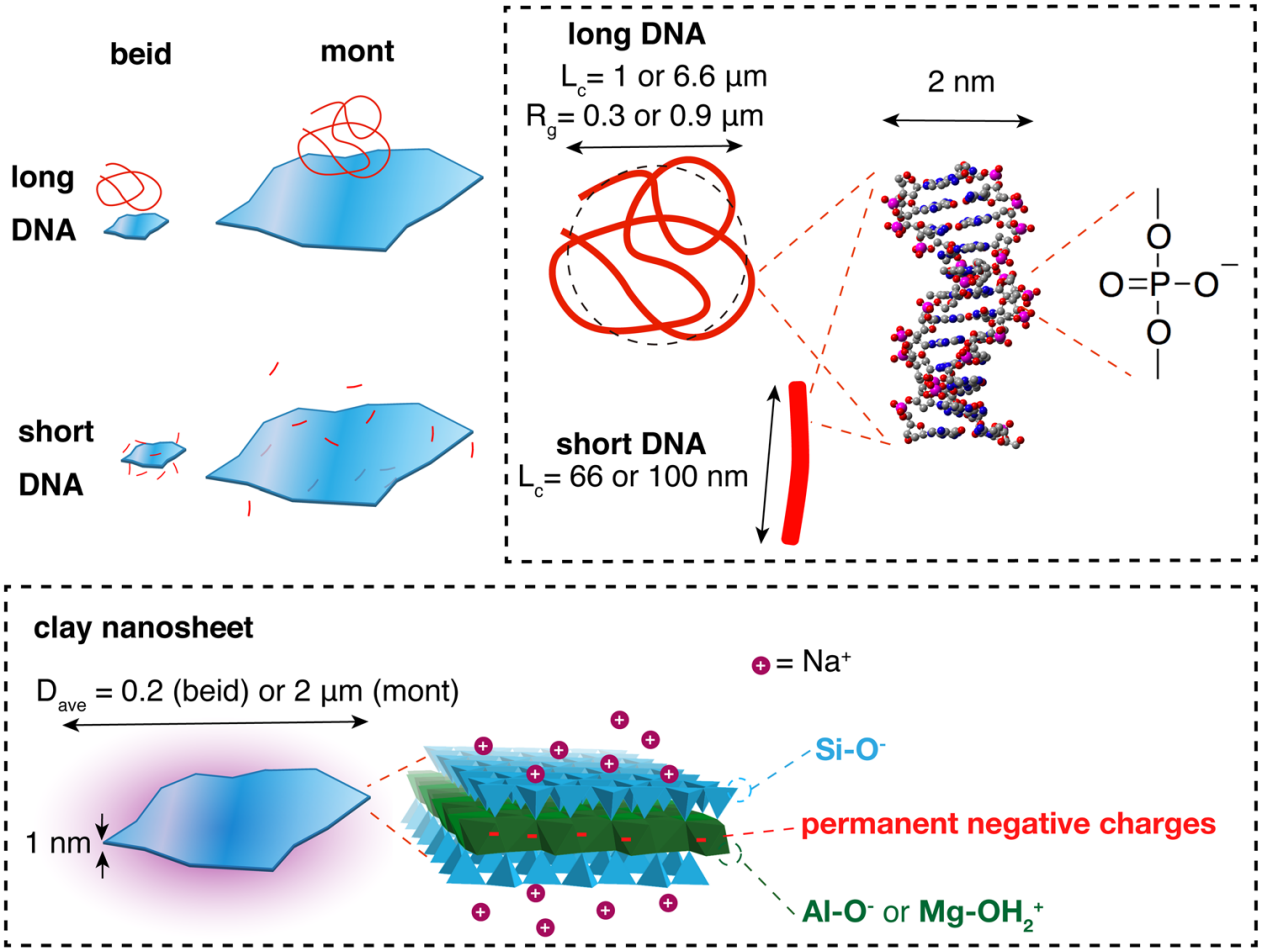
Schematic representations of the various (clay, DNA) systems investigated as described in the Materials section. The two clay minerals were either Na-montmorillonite (mont) or Na-beidellite (beid) which are 2:1 clays with two silicate tetrahedral sheets and a central octahedral alumina sheet. Isomorphic substitutions in the octahedral sheet (montmorillonite) or the tetrahedral sheets (beidellite) are compensated with hydrated Na^+^ cations concentrated near the sheets.

Here, we report on the formulation and physical study of two-component colloidal suspensions comprised of anionic clays and double stranded DNA. Various (clay, DNA) systems differing by the nature of the clay (montmorillonite or beidellite ≈1.0 nm thick plate-like clays with diameters around 2 μm and 0.2 μm, respectively) and the length of the DNA (long and short) were prepared (Fig. 1). The smectite clays belong to the family of 2:1 clays with silicate tetrahedral sheets sandwiching an octahedral alumina sheet. Furthermore, isomorphic substitutions in either the octahedral sheet (montmorillonite clay) or the tetrahedral sheets (beidellite clay) renders them anionic with hydrated cations (e.g. Na^+^ or Li^+^) concentrated near the sheets^34^. In spite of the DNA and clay nanosheets being both negatively charged, we found that the DNA adsorbs to some extent on the clay sheets. We also found that the 2-component suspensions generally demix into a clay-poor isotropic phase and a clay-rich nematic phase, which corresponds to a large widening of the I/N biphasic region. We argue that this is due to the appearance of attractive, DNA-mediated, interactions between the clay nanosheets, inhibiting their swelling.

## Materials and Methods

Different (clay, DNA) systems were prepared with two kinds of natural clay, beidellite and montmorillonite, and four kinds of DNA to test the generic character of our observations.

### Materials

Natural beidellite (SBId-1) was purchased from the Clay Minerals Society. It was purified according to procedures described in detail elsewhere^35^. In short, the clay was first suspended three times in a LiCl 1 M solution. After dialysis, impurities were removed by sedimentation and size selection was performed by centrifugation at various speeds. The sample used in the present study corresponds to the smallest size fraction of beidellite. Previous characterization by transmission electron microscopy (TEM), small-angle X-ray scattering (SAXS), and cation exchange capacity (CEC) measurements yielded respectively an average diameter around 200 nm (Figure SI1a), a nanosheet thickness of 0.7 nm, a density of 2.6 g.cm^−3^, and a CEC of 90 meq/100 g^35^.

The natural Na-montmorillonite (Kunipia-F from Tsukinuno, Japan) was supplied from Kuninine Industries via the Clay Mineral Society of Japan. It was purified before use by centrifugation. The montmorillonite sheets have a lateral size of several µm and the thickness of ~1 nm, as confirmed by atomic force microscopy (Figure SI1b). The CEC is 119 meq/100 g and the density is 2.7 g.cm^−3^.

For the (beidellite, DNA) systems, we used either salmon or herring sperm DNA. Low molecular weight salmon sperm d.s. DNA in freeze-dried powder form was purchased from Sigma Aldrich (CAS Number: 100403-24-5) and used without further purification. This DNA is on average 200 base pairs (b.p.) long and since the DNA stacking period is 0.332 nm, the DNA strand is on average 66 nm long; it is called “short DNA” hereafter. High molecular weight herring sperm d.s. DNA in freeze-dried powder form was also purchased from Sigma Aldrich (CAS Number: 438545-06-3) and used without further purification. This DNA is highly polydisperse and ranges from 1000 to 10^4^ b.p.; it is therefore from 330 to 3300 nm long and is called “long DNA” hereafter.

For the (montmorillonite, DNA) systems, we used the salmon sperm d.s. DNA supplied from Maruha Nichiro Corporation as received since it was already purified by the company. Two kinds (“short DNA” and “long DNA”) of DNA samples with different average molecular weights, 300 b.p. and 2 × 10^4^ b.p. were used. The 300 b.p. DNA was prepared from the 2 × 10^4^ b.p. one by using a splitting restriction enzyme. The purity of the samples was checked with UV spectroscopy and by gel electrophoresis. The 300 b.p. DNA is 100 nm long whereas the 2 × 10^4^ b.p. one is 6.6 µm long.

DNA solutions of suitable concentrations were prepared by direct dissolution in deionized water under gentle stirring for about an hour.

### Sample production

**Figure 2:**
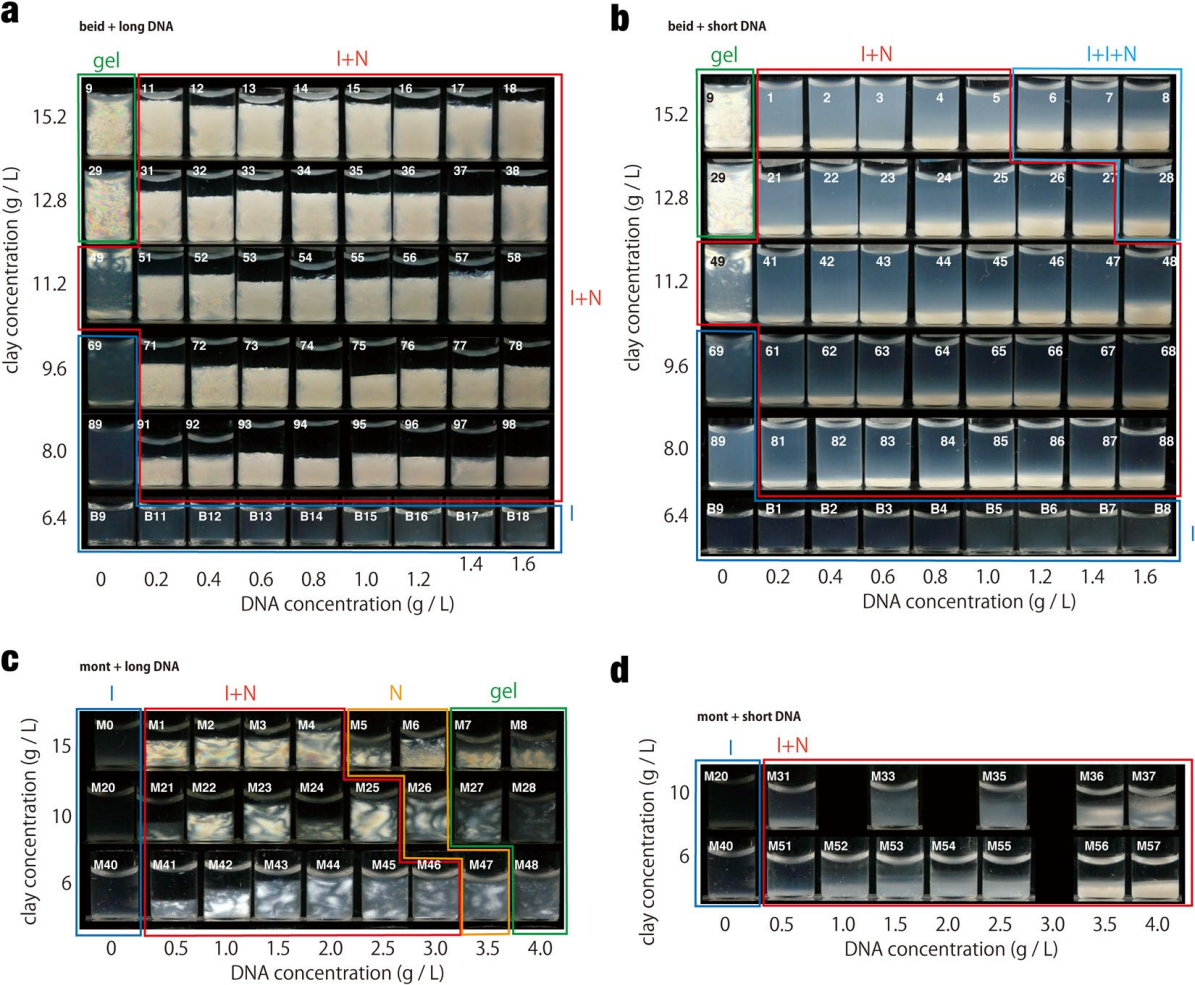
Photographs of samples of clay-DNA mixtures in vials viewed between crossed polarizer and analyser. (**a**) (beid, long DNA) system; (**b**) (beid, short DNA) system; (**c**) (mont, long DNA); and (**d**) (mont, short DNA) systems.

**Figure 3:**
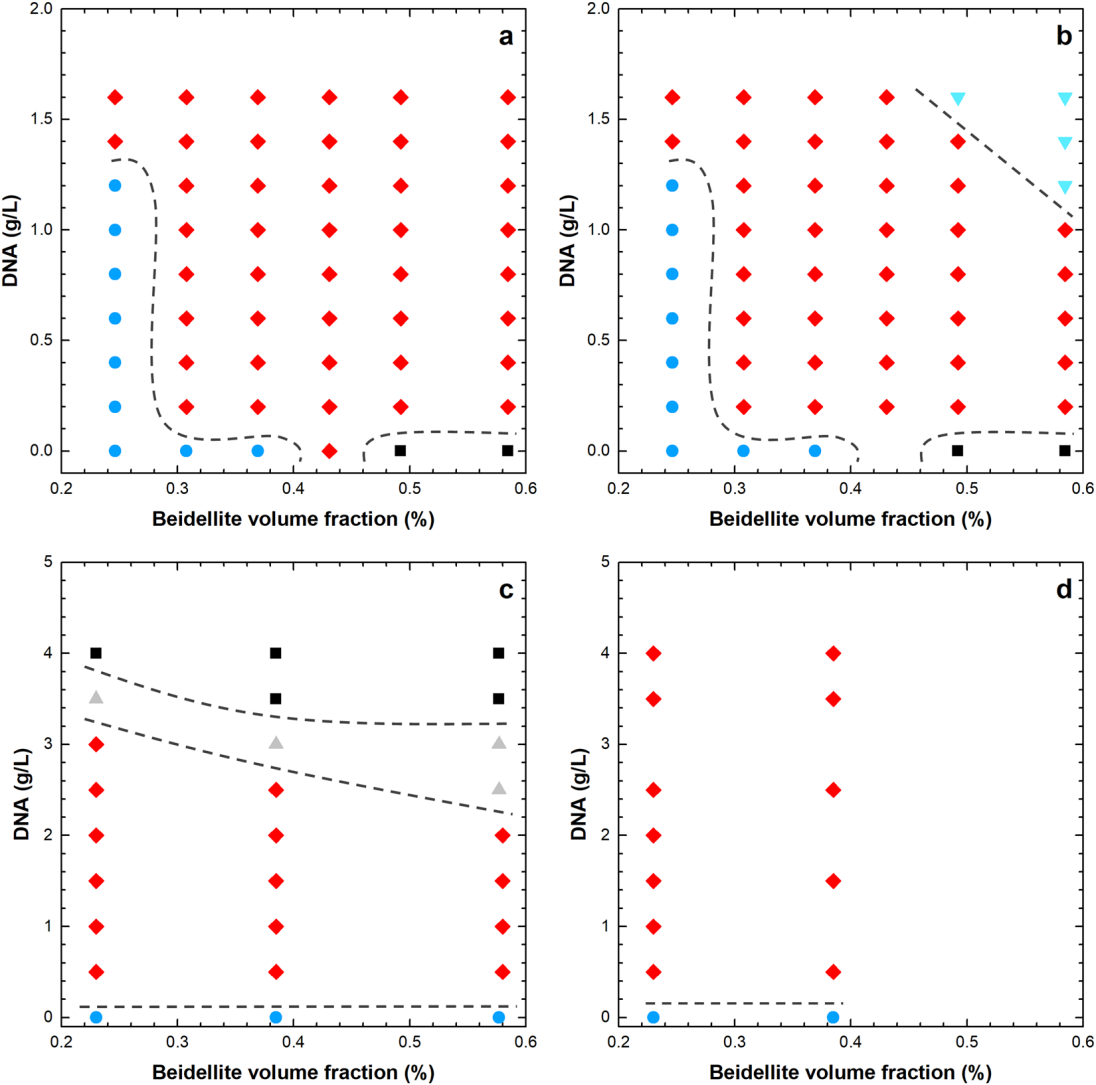
Phase diagrams of (**a**) (beid, long DNA) system; (**b**) (beid, short DNA) system; (**c**) (mont, long DNA) system; (**d**) (mont, short DNA) system. The symboles indicate: (Filled blue circles) isotropic, (filled red diamonds) isotropic + nematic, (gray triangles) nematic, (cyan triangles) isotropic + isotropic + nematic samples, and (black squares) the samples in the gel state.

Samples of (clay, DNA) aqueous suspensions of known clay and DNA concentrations were prepared by directly mixing the required volumes of pure clay (at initial concentration ranging from 8 to 22 g/L and ionic strength of 10^−4^ M NaCl for montmorillonite and LiCl for beidellite) and DNA dispersions (at initial concentration ranging from 1 to 8 g/L) in 4 mL glass vials. The final ionic strength (mostly due to NaCl impurities brought by the DNA) of the mixtures varies with increasing DNA content. It ranges between 10^−4^ M and 10^−3^ M for the montmorillonite and (beidellite – long DNA) systems and can reach up to 5 × 10^−3^ M for the (beidellite – short DNA) systems. The sample compositions were defined so as to appropriately explore the 2-dimensional phase diagram of the suspensions as a function of clay and DNA concentrations (see Figs 2 and 3 for clay and DNA concentrations of the mixtures studied). All observations were carried out at room temperature.

### Phase assessment

Series of samples in glass vials were first visually inspected both in natural light to check sample homogeneity and, when relevant, the number of coexisting phases, and between crossed polarizers to detect phase birefringence. The sample series were photographed with a digital camera. Sample gelation was simply detected by tilting the vials.

### UV spectroscopy

UV spectroscopy was performed in the 200–350 nm wavelength range using an Agilent Cary 5000 spectrophotometer. Series of samples of known DNA and clay concentrations were prepared to record calibration straight lines and to measure the absorption coefficients of clay and DNA (short DNA (Aldrich): 0.0465 (g/L)^−1^; long DNA (Aldrich): 0.0483 (g/L)^−1^; short DNA (Maruha Nichiro): 0.0590 (g/L)^−1^; long DNA (Maruha Nichiro): 0.0488 (g/L)^−1^; beid: 0.452 (g/L)^−1^; mont: 0.787(g/L)^−1^). Samples were diluted enough (typically by a factor 100) to reach the linear regime of the spectrophotometer and were transferred into 2 mL plastic vials of 10 mm optical path.

### Small-angle X-ray scattering

SAXS experiments were carried out at the SWING beamline of the SOLEIL synchrotron radiation facility (Saint-Aubin, France). Measurements were made using a fixed energy of 12.0 keV and a sample-to-detector distance of 6.56 m; the pixel size was 42 µm. The typical accessible range of scattering vector modulus *q* was 1.6 × 10^−2^ – 1.6 nm^−1^ (*q* = (4π/λ)sin θ, where 2θ is the scattering angle and *λ* = 0.1033 nm is the wavelength). Scattering patterns were recorded on an AVIEX 170170 CCD camera formed by four detectors and placed in a vacuum detection tunnel. The scattering patterns were radially averaged to obtain the scattering curves *I*(*q*).

### X-ray diffraction

X-ray diffractograms (XRD) were obtained using a Bruker D8 Advance diffractometer on films obtained by drying the samples on glass slides. The wavelength of the monochromatic X-rays was 1.54 Å (Cu Kα) and a 2θ range from 0.5 to 15 ° was explored.

## Results

The addition of DNA, even at very low concentration (~0.2 g/L), to beidellite clay suspensions quickly gave rise to a phase separation into two phases (Fig. 2a,b). The visual inspection of samples in glass vials shows that the bottom phase is cloudy and birefringent whereas the top phase is transparent and isotropic. Moreover, the top phase only displays little flow birefringence, which means that it is poor in clay. This phase separation therefore seems different from the usual isotropic/nematic phase coexistence of pure clay suspensions where both phases have almost the same concentrations due to the small width of the I/N biphasic region, leading to strong flow birefringence of the top isotropic phase. The relative proportion of the bottom phase is small but increases with DNA concentration for the (beid, short DNA) system (Fig. 2b) whereas it is large but seems to remain more or less constant for the (beid, long DNA) one (Fig. 2a). Moreover, the proportion of bottom phase also increases with the clay concentration, as intuitively expected. In addition, a few samples, for example in the (beid, short DNA) system at high clay and DNA concentrations, also displayed three coexisting phases, the upper two being transparent and isotropic. Apart from this latter feature, these observations seem to be fairly generic since the (mont, DNA) systems behave in quite a similar way for both short (Fig. 2c) and long DNA (Fig. 2d).

Considering this recurrent phase separation and the turbidity of the bottom phase, one may wonder whether adding DNA to aqueous clay suspensions does not simply lead to their flocculation. However, the bottom phase, in the glass vials, does not really look like a precipitate. Its closer inspection, in samples filled into flat glass capillaries, by polarized light microscopy, usually display a featureless birefringent material but sometimes clearly reveals typical nematic threaded textures (Figure SI2). Moreover, SAXS results (see below) show that the clay nanosheets are about 40 nm apart in the bottom phase, in contrast to usual clay flocculates where typical interparticle distances are smaller than 10 nm^36^.

Our visual observations of samples in test-tubes are summarized in phase diagrams (Fig. 3a–d) that look fairly simple but are actually rather unexpected since it appears that a very small addition of DNA to clay suspensions leads to phase separation, whatever the clay concentration. Moreover, mixtures showing only the pure nematic fluid phase are no longer observed, even though single-phase nematic gel samples were found at higher concentrations (Fig. 3c, mont, long DNA).

**Figure 4:**
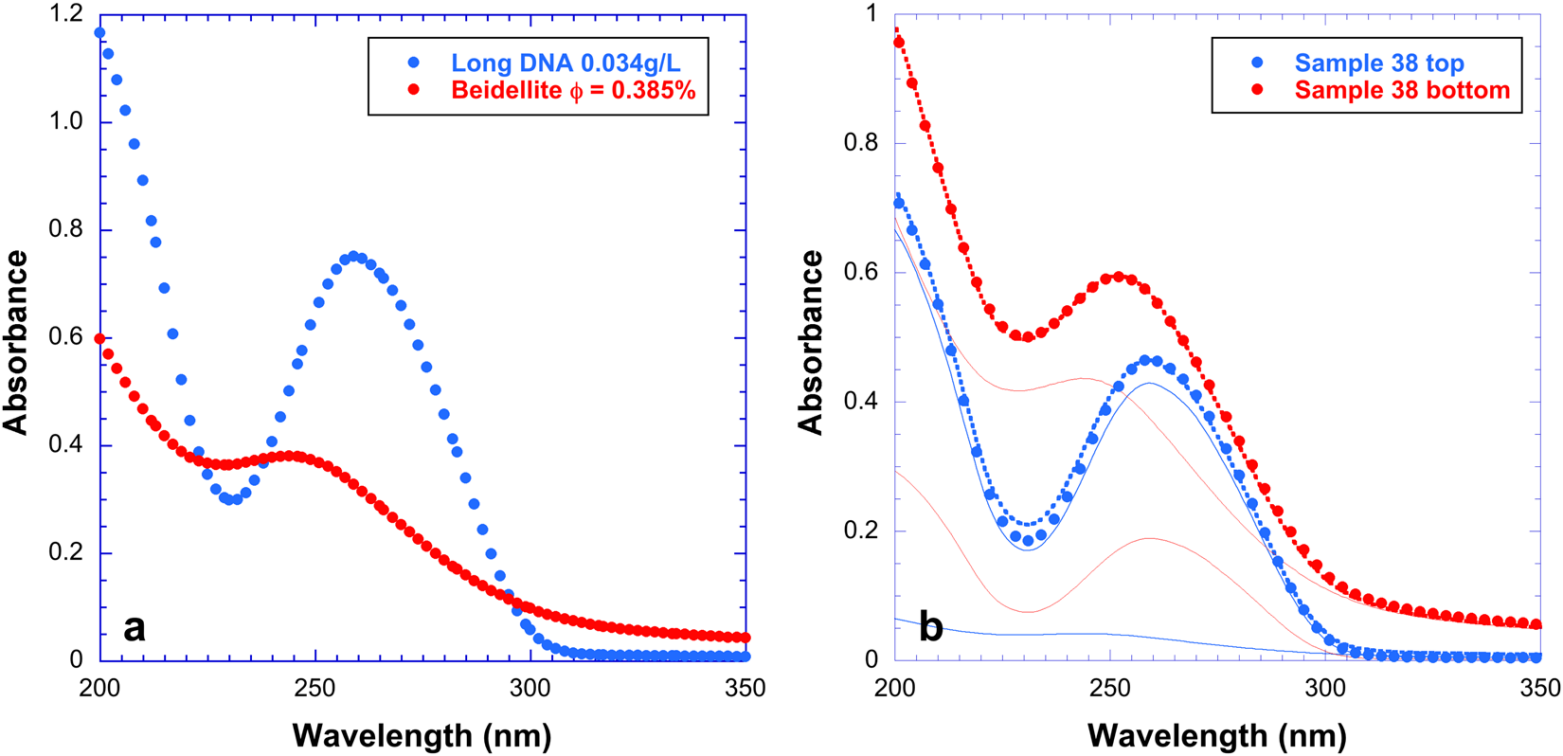
(**a**) UV spectra of a pure beidellite suspension (volume fraction: 0.385%) and of a pure long DNA solution (0.034 g/L). (**b**) Example of decomposition of UV spectra for one sample (top and bottom phases of sample 38 consisting of a Beidellite clay (12.8 g/L)/long DNA (1.6 g/L) mixture). See Fig. 2 for more information.

**Figure 5:**
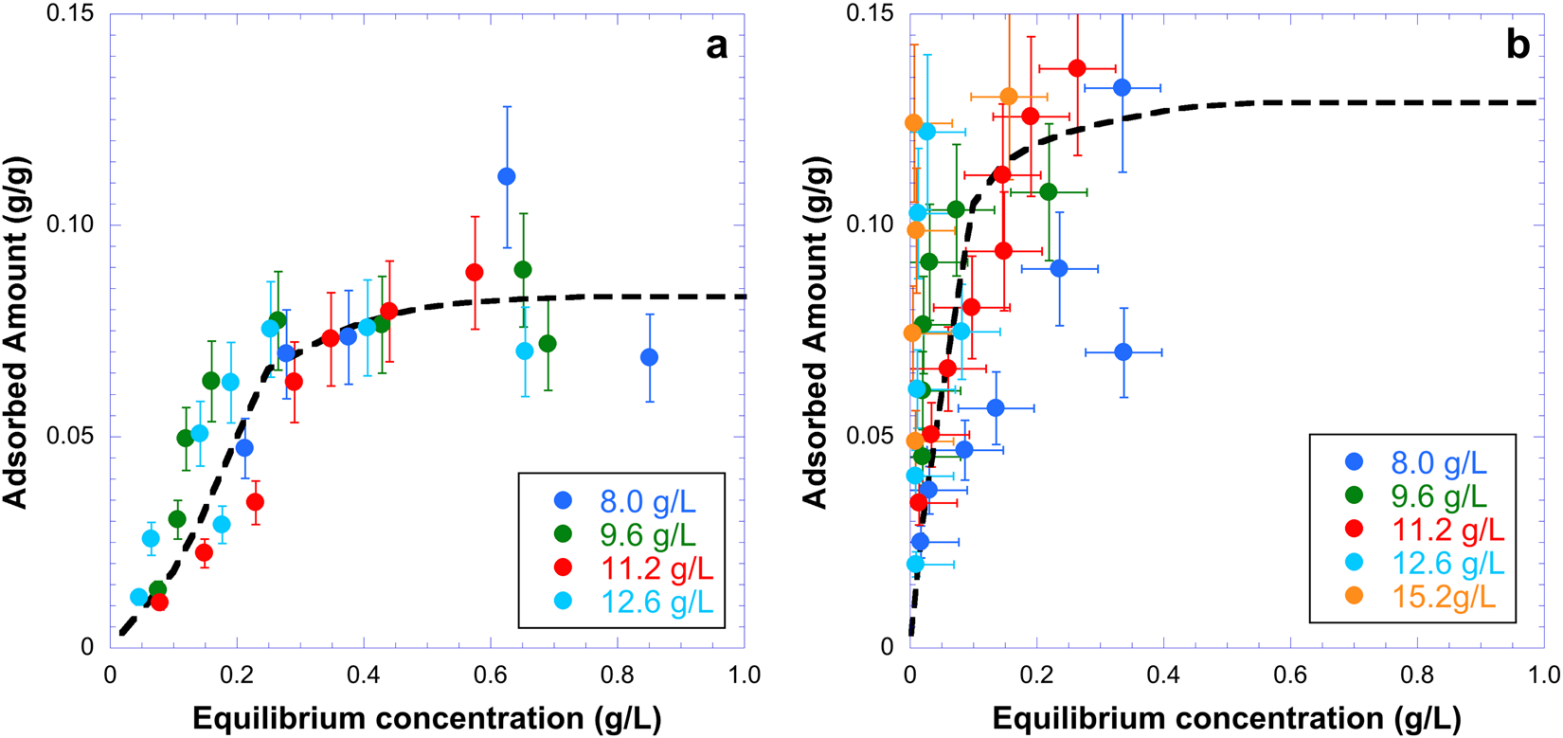
Adsorption isotherms of DNA on beidellite for (**a**) long DNA; (**b**) short DNA. Clay concentrations are denoted in the boxes. (Lines are only guides to the eye).

UV-absorption spectroscopy experiments were carried out to further investigate the nature of the interaction between DNA and the clay nanosheets. In the present case, to keep sample integrity, liquid/solid separation was not carried out as would be done for classical adsorption isotherm measurements. To determine retention curves of DNA on clay particles, we then analyzed both the top and bottom phases when present (see Fig. 2) by UV spectrometry. Pure beidellite clay aqueous suspensions display a broad absorption peak with a maximum at a wavelength of 245 nm whereas pure DNA solutions display a broad peak at 260 nm (Fig. 4a). All absorption spectra were then fitted by a linear combination of the absorption signals of clay and DNA suspensions. Figure 4b shows an example for sample 38, with 12.8 g/L beidellite and 1.6 g/L long DNA, (Fig. 2a, second row). Still for sample 38, the top phase (poor in clay) has 1.27 g/L of “free” DNA and the bottom phase (rich in clay) has 0.65 g/L of “free” DNA. Both amounts of “free” DNA are lower than the initial one (1.6 g/L), which reveals the occurrence of adsorption in the system. Considering the clay concentration in each phase, derived from the UV spectra, and the difference between initial and final DNA concentrations, the amount of DNA adsorbed per gram of clay can then be determined in each phase. Finally, taking into account the relative phase proportions determined by visual inspection of the samples (Fig. 2), the adsorbed DNA amount and the average “free” DNA concentration were calculated for each sample. The data thus obtained can be regarded as corresponding to retention isotherms and are plotted in Fig. 5 for beidellite clay for long (Fig. 5a) and short (Fig. 5b) DNA. In spite of the large error bars in the isotherm induced by the complex data treatment, general trends can be derived. For both the short and long DNA, retention isotherms sharply increase at low equilibrium concentration before levelling off at higher concentration. The isotherms are almost independent of clay concentration, which strongly suggests that adsorption *sensu stricto* is the main retention mechanism in this case. The isotherms are sharper and level off at a higher retained amount in the case of the short DNA, which suggests that this DNA has slightly higher affinity towards beidellite. Furthermore, the shape of the curves are close to those obtained in previous studies on nucleotide adsorption on clay minerals^13,14^, where it was evidenced that the main adsorption mechanism was phosphate complexation on the edge OH groups of clay minerals. It then appears reasonable to assume a similar adsorption mechanism for both DNAs in the present case.

The SAXS patterns of the bottom and top phases of all the samples were recorded to try to confirm the presence and organization of the clay nanosheets. The scattering by the clay is the major contribution to the SAXS signal since all clays have very good contrast with water and since the scattering by pure DNA at such low concentrations cannot be easily detected in our experimental conditions.

**Figure 6:**
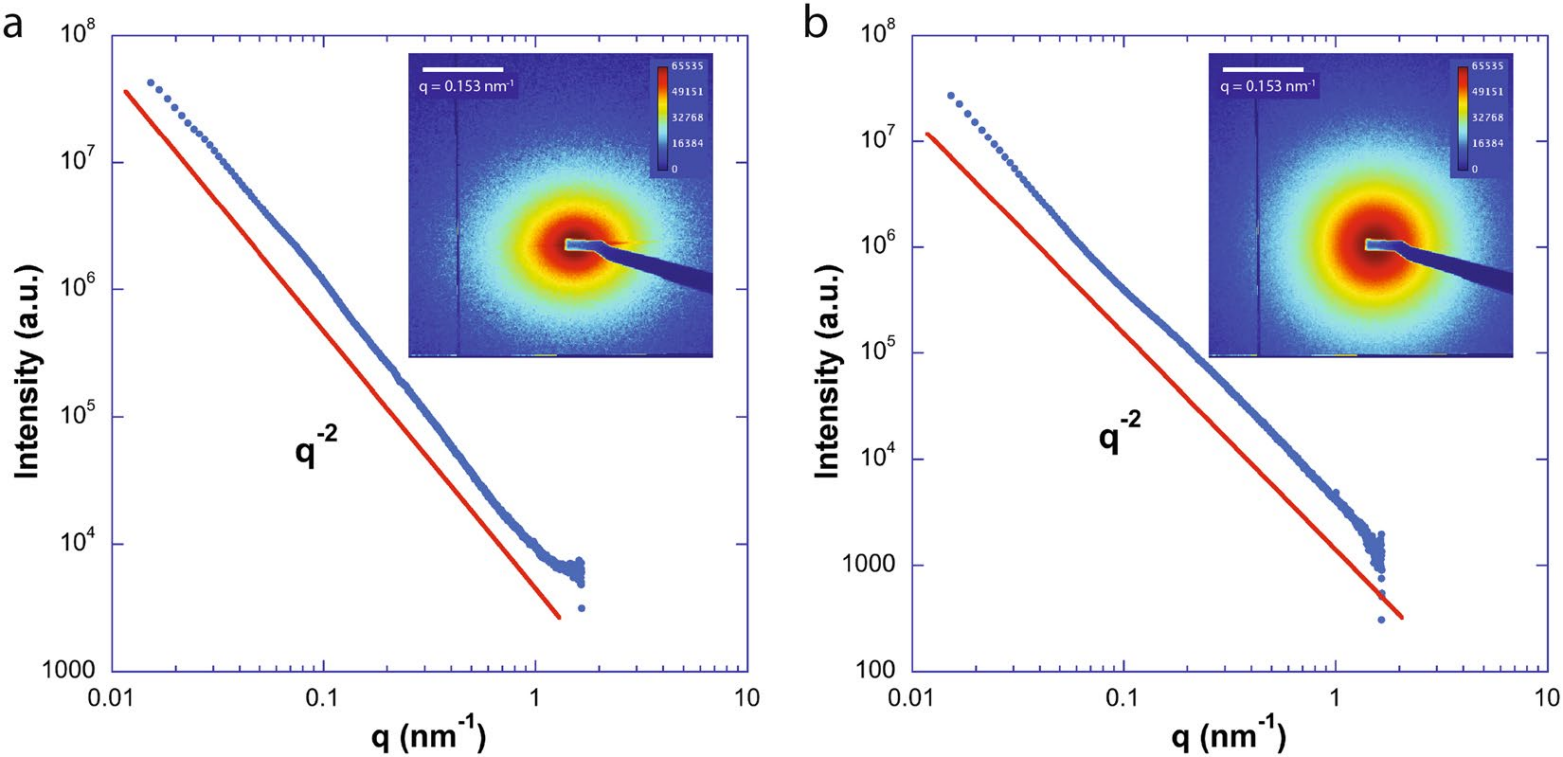
*I*(*q*) curves obtained by radial integration of the SAXS patterns (insets) of pure beidellite and pure montmorillonite at a volume fraction of 0.2%.

The SAXS patterns of pure clay suspensions display strong scattering signals. For isotropic dilute clay suspensions, this scattering is simply described by the nanosheet form factor averaged over all orientations in *q*-space, which gives rise to a *q*^−2^ dependence of the scattered intensity in the explored *q*-range (Fig. 6)^31^. However, nematic concentrated suspensions usually show diffuse scattering peaks at *q* which are in ratios 1:2:3:4 … because they arise from a 1-dimensional stacking liquid-like order of the clay sheets, with a period *d*. As for lamellar phases of surfactants^37^, this period *d* is related to the clay volume fraction, *φ*, by *d* = *δ/φ* where *δ* ~ 0.7 nm is the nanosheet thickness^31^. This scattering feature shows that a strong lamellar short-range order prevails in the nematic phase of clay suspensions.

**Figure 7:**
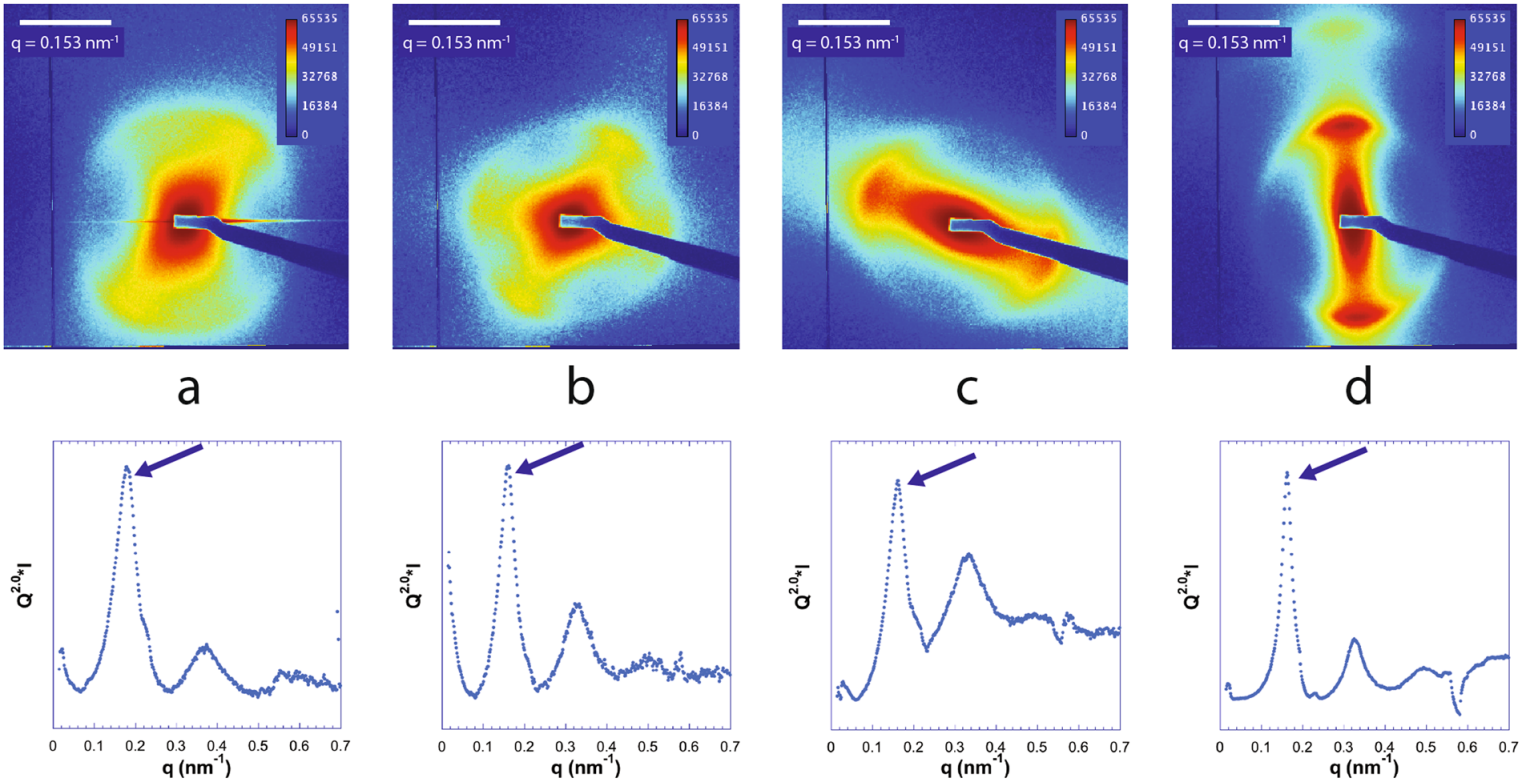
Examples of SAXS patterns and associated *I*(*q*) curves for some nematic phase samples of the (beid, long DNA) system. (**a**) Sample 76; (**b**) Sample 56; (**c**) Sample 36; (**d**) Sample 16. (See Fig. 2a for sample identification).

**Figure 8:**
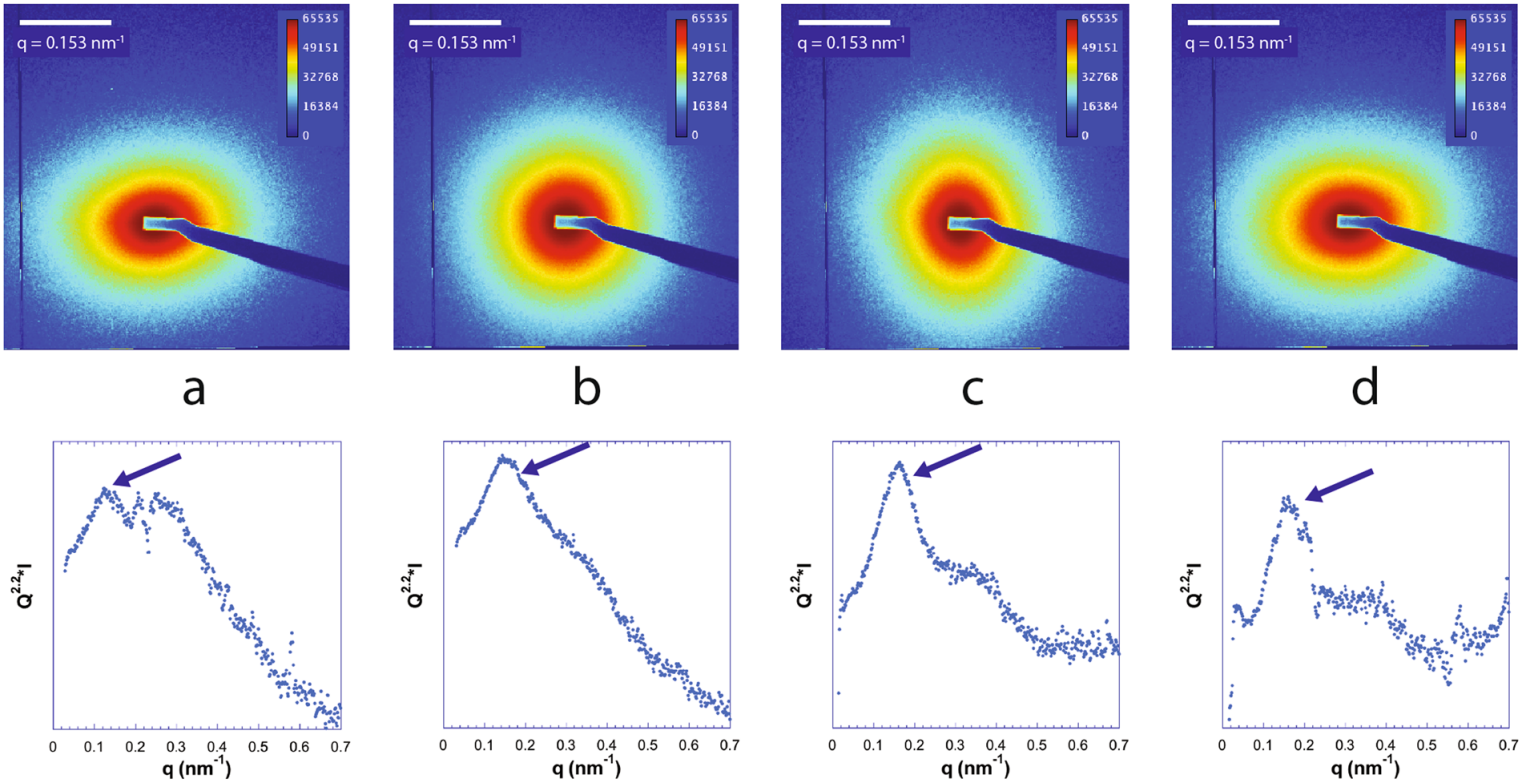
Examples of SAXS patterns and associated *I*(*q*) curves for some nematic phase samples of the (beid, short DNA) system. (**a**) Sample 86; (**b**) Sample 66; (**c**) Sample 46; (**d**) Sample 26. (See Fig. 2a for sample identification).

**Figure 9:**
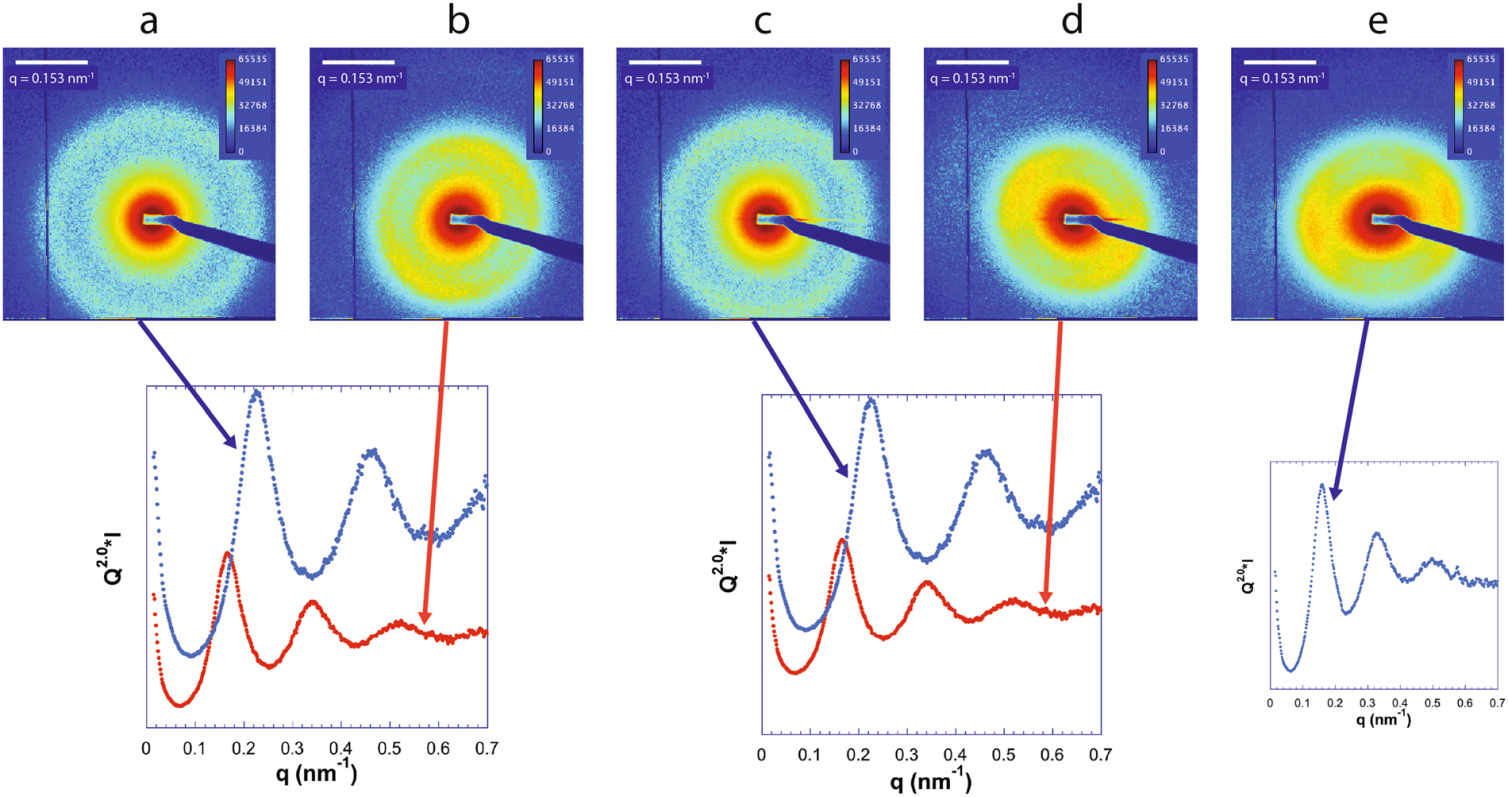
Examples of SAXS patterns and associated *I*(*q*) curves for some samples of the (mont., DNA) systems. (**a**) Sample M55 (short DNA); (**b**) Sample M45 (Long DNA); (**c**) Sample M35 (short DNA); (**d**) Sample M25 (Long DNA); (**e**) Sample M5 (long DNA). (See Fig. 2c and d for sample identification).

**Figure 10:**
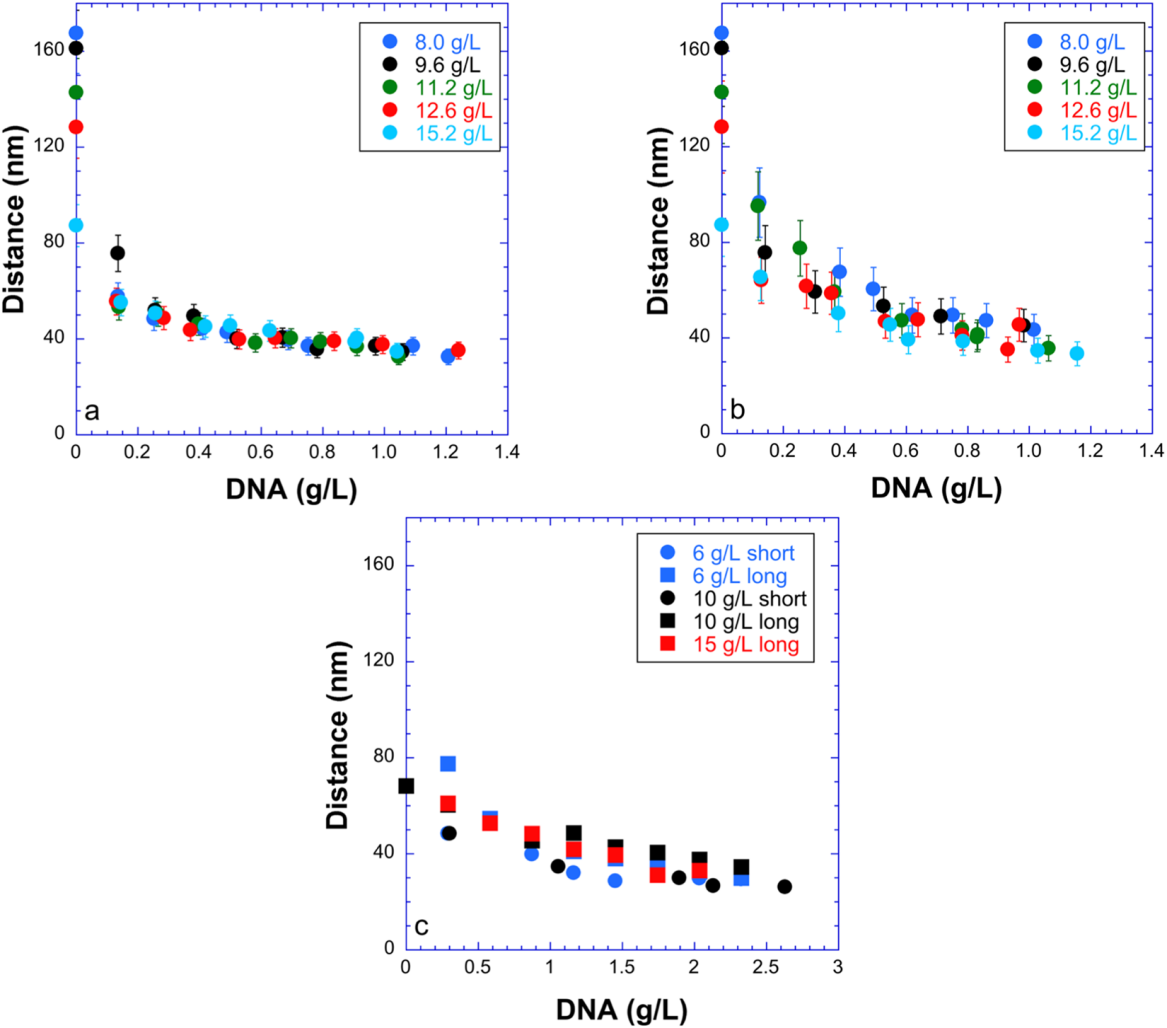
Evolution with the amount of DNA of the interparticle distance measured on the SAXS patterns. (**a**) (beid, long DNA) system; (**b**) (beid, short DNA) system; (**c**) (mont, DNA) systems.

In general, in our (clay, DNA) systems, the top phase only showed weak and featureless scattering which must be due to the few clay sheets remaining in the top phase. In contrast, the bottom nematic liquid crystal phase always gave rise to a strong SAXS signal (Figs 7–9), which often displayed diffuse liquid-like scattering peaks (with peak widths increasing with increasing *q*), as observed with pure clay suspensions. In the case of the (beid, long DNA) system (Fig. 7), peaks are well defined whereas for the (beid, short DNA) ones (Fig. 8), the peaks are much broader and weaker. In contrast, for the montmorillonite clay system with about ten times larger diameter plates compared to beidellite clay plates (≈2000 nm versus ≈200 nm), the SAXS liquid peaks of the nematic phase are well defined for both long DNA (Fig. 9, images b, d, and e, for samples M45, M25, and M5 respectively) and short DNA (Fig. 9, images a and c for samples M55 and M35, respectively). The positions of the diffuse scattering peaks were obtained from the *I*(*q*) scattering curves in Kratky representation (*q*^2^*I*(*q*) vs *q*). As mentioned above, this provides us with the average distance between the clay nanosheets (average stacking period) in the bottom phase for all samples. This average distance is plotted versus DNA concentration for all samples of the (beid, long DNA) systems in Fig. 10a. The first striking observation is that the bottom phase is not a plain precipitate of clay nanosheets almost at contact (i.e. due to van der Waals attractions between sheets) because this would lead to a stacking period of only a few nanometers^36^. The typical stacking periods measured in the bottom phase are in the 30–160 nm range as the DNA concentration is decreased, which instead suggests a concentrated nematic suspension where the nanosheets still strongly repel each other. This conclusion is consistent with the clear nematic textures sometimes detected in polarized-light microscopy (Figure SI2). The second striking observation is that, beyond a DNA concentration of ~0.3 g/L, the clay stacking period no longer follows the usual *d* = *δ/φ* law but it becomes completely independent of the clay concentration. The only effect of increasing the clay concentration is then to increase the proportion of the bottom phase at the expense of the supernatant (Fig. 2). Moreover, at constant clay concentration, the clay stacking period only weakly decreases with increasing DNA concentration. These unexpected observations were made for all (clay, DNA) systems investigated (Fig. 10b,c).

## Discussion

The most salient consequence of adding DNA, whatever its size, even in small amounts (0.2 g/L), to natural clay suspensions is to induce phase separation. The bottom phase seems to remain nematic and therefore the main effect of DNA addition is to widen the I/N biphasic region. Moreover, in this region, the tie lines link a clay-poor isotropic phase to a clay-rich nematic phase. This agrees with the fact that upon increasing DNA overall concentration the clay stacking period in the bottom phase decreases from an initial swollen state (between ≈80 nm and 160 nm) to values that level off between ≈40 nm and 30 nm. Such evolution suggests the appearance of a new attractive interaction mediated by the DNA, which suppresses swelling of the clay sheets and is consistent with the observation of macroscopic phase separation. The intensity of this interaction should be of the same order of magnitude as that of the electrostatic repulsions since the equilibrium distance between the particles is indeed affected but not to the point that the particles are almost brought to contact, as in flocculation.

Several of these experimental facts, like the widening of the I/N biphasic gap and the increased volume fraction of the nematic phase, would suggest the appearance of attractive depletion interactions between the clay nanosheets upon DNA addition. However, an order of magnitude calculation of the depletion pressure (see Supplementary Information for details) shows that it should remain negligible in front of that, ~500 Pa^35^, due to the electrostatic repulsions in all of our various experimental (clay, DNA) systems. Such simple depletion calculations^22^, though, do not account for electrostatic or many-body effects that could become important for the highly anisotropic charged clay nanosheets. They also do not consider depletion effects on nanosheet stacks. Another serious objection to the relevance of depletion interactions is that, based on the determination of the adsorption isotherms, the DNA cannot be considered as a non-adsorbing polymer for the clay sheets, even though both species are negatively charged.

It is in fact likely that less generic and more system-specific effects govern the physical chemistry of the (clay, DNA) colloidal dispersions. Indeed, previous studies have shown that nucleotides strongly adsorb on the particle rims through phosphate/metal bonds^13,14^. Moreover, we note that the DNA/clay association occurs rapidly and irreversibly, which is the sign of strong interactions much larger than thermal energies. The shape of the adsorption isotherms suggests that, upon increasing DNA concentration, the first DNA molecules (up to ~0.3 g/L) adsorb and wrap onto the nanosheet rims. At the same time, a DNA-mediated sharp drop in average nanosheet separation is observed. Then, further addition of DNA does not result in adsorption and does not affect the average nanosheet separation, which can be interpreted as the DNA mostly partitioning towards the clay-poor top phase.

According to the DNA length, several different mechanisms may induce an indirect, DNA-mediated, steric interaction between the nanosheets. Upon system preparation, long DNA molecules could quickly and irreversibly adsorb onto the rims of different clay nanosheets, thus bringing them closer together than they would if they only experienced electrostatic repulsions. Because of the very long contour length, up to ≈6.6 microns, a single DNA chain can adsorb onto sections of rims of *multiple* plates simultaneously tethering and bringing them to close proximity (i.e. forming a plates-on-a-string structure). In this scenario the closest distance between plates is expected to be of order the persistence length of DNA between 100 nm (low salt ≈few mM) and ≈50 nm (at high salt ≈0.5 M), because significantly smaller inter-plate separations would lead to DNA bending penalties. Indeed, the measured levelled off inter-plate distance ≈40 nm to ≈35 nm is within a factor of two of the persistence length of double stranded DNA.

**Figure 11:**
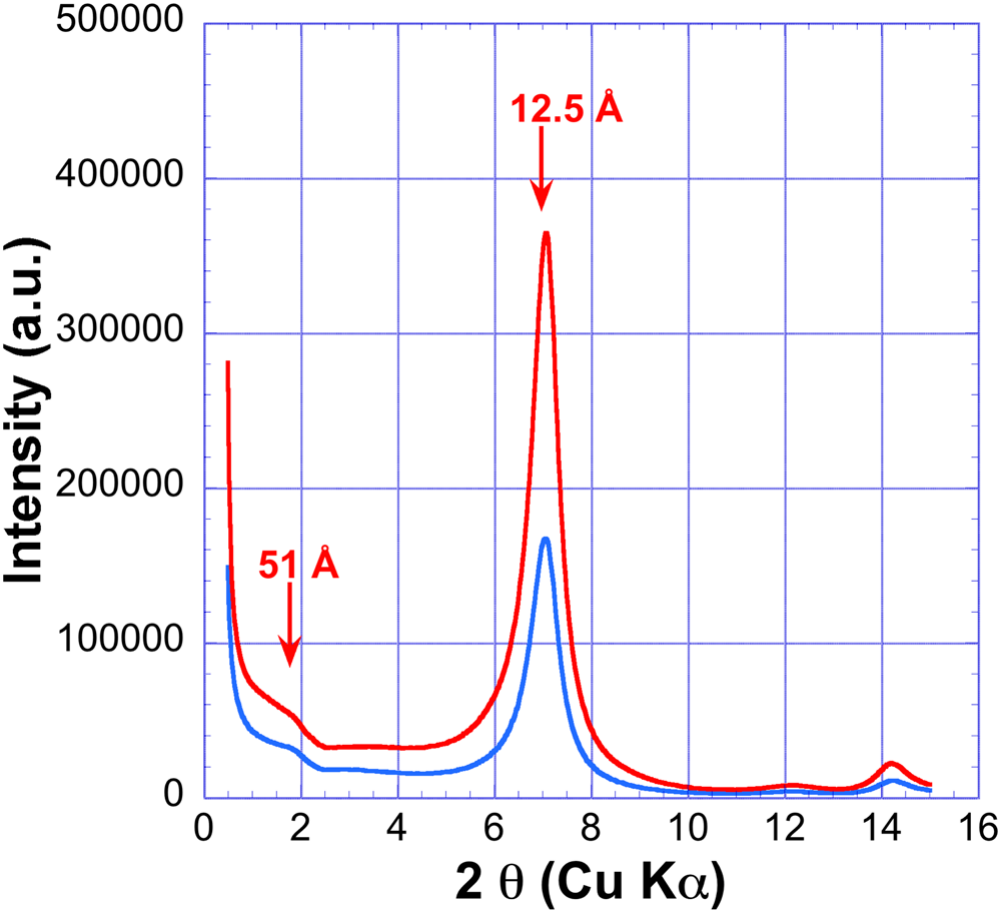
X-ray diffractograms obtained after drying of samples 16 and 26. (See Fig. 2 for sample identification).

The assumption of the long DNA bridging conformation is further confirmed by the XRD patterns obtained upon drying beidellite/DNA samples. The patterns exhibit, in addition to the classical peak corresponding to the one-layer hydrate of Na-Beidellite at 1.25 nm, an additional signal corresponding to larger distances, around 5 nm (Fig. 11). This larger period is strongly supportive of our long DNA bridging model and could indeed arise from bridging portions of DNA chains trapped between the clay nanosheets: upon drying, DNA chains within the plane of the sheet are expected to bend on the scale of DNA’s persistence length, thus producing the additional XRD shoulder at ≈5 nm.

For the case of short DNA, for both the beid (Fig. 10b) and mont (Fig. 10c) clay systems, we observe that the intersheet distance levels off between 30 nm and 40 nm with increasing DNA concentration. We note that for short DNA the relevant length scale is the contour length because it is of order the persistence length of DNA (~100 nm in our low salt conditions). Thus, the short DNA used in this study may be viewed as slightly bent rods of length ≈66 nm to 100 nm and diameter 2.5 nm (for hydrated B-DNA) (see Fig. 1). Our measurement of 30–40 nm intersheet distance is consistent with tethering (bridging) of neighboring clay sheets with sections of each short DNA rod (about half) used up by adsorption to two neighbouring clay rims.

A semi-quantitative analysis of the adsorption limit of 0.1 g DNA per g of clay (Fig. 5) also somehow further supports the bridging mechanism: The total clay edge length can be estimated to 1.1 × 10^10^ m per g of clay whereas, for 0.1 g DNA, the adsorbed DNA length is 3.1 × 10^10^ m. Although these two values are of the same order of magnitude, they do not quite agree. This discrepancy could simply be due to the fact that the beidellite nanosheets are neither monodisperse nor perfect circular discs. However, this rough calculation of the adsorption limit does not consider the bridging and looping portions of DNA that are not actually adsorbed on the clay rims but are nevertheless trapped close to the clay nanosheets and still contribute to the DNA amount associated with the clay. The discrepancy between the two values may then be due to the DNA bridges and loops.

Another possible mechanism would be linked to electrostatic interactions. Indeed, DNA addition and adsorption lead to both a higher (negative) electric charge on the particles and an increase in ionic strength (from ~10^−4^ M to ~a few 10^−3^ M for 1 g/L DNA). The latter effect alone can hardly explain the phase separation and the smaller interparticle distances since they are not observed on pure clay suspensions at such ionic strength. However, the increase in both particle charge and ionic strength might give rise to Ise-Sogami type attractive interactions^38–40^. Such electrostatic mechanism has, to our knowledge, only been rarely reported for monovalent counter-ions^41^. Therefore, much further work is required to assess the validity of this mechanism.

Another very unusual and yet unexplained feature of these colloidal mixtures is that, after DNA adsorption is completed (beyond 0.3 g/L), the average distance between the nanosheets in the bottom phase is almost independent of the overall clay concentration. This feature is particularly marked for the (beid, long DNA) system (Fig. 10a). This means that most points in the biphasic region demix into a nematic phase of constant clay concentration. In other words, the nematic branch of the binodal line in the phase diagram must be almost vertical. Due to sample gelation at high clay and/or DNA concentrations, however, this branch could not be readily observed by visual inspection of the samples.

The average distance between the nanosheets in the bottom phase being independent of the overall clay concentration may shed some light on the conformation of the DNA in these systems. Our adsorption experiments showed that DNA adsorb on the rims of the clay sheets. There are two types of conformations that DNA will adopt in its interactions with the clay edges: First, a bridging conformation where a single DNA links neighboring clay sheets by adsorbing to sections of their respective rims, therefore producing an attractive interaction. Second, a DNA chain can also form a “looping” conformation between two sections adsorbed on the same clay rim (i.e. producing a clay sheet with one or more attached DNA loops protruding away from the rims). These loops are a source of repulsion between the clay sheets and most importantly they can be quite long-ranged, depending on the length of the DNA loop. In this model, the spacing arising from the balance of repulsions (DNA looping) and attractions (DNA bridging) could be dependent on the concentration of DNA only and independent of the clay sheets, in agreement with the experiments. This is because DNA is simultaneously providing the attractive and repulsive forces, with the magnitudes of the repulsive and attractive forces being proportional to the fraction of “looping” and “bridging” conformations, respectively. This model is inspired by the conformations observed for double-end-anchored poly(ethylene oxide) chains forming looping and bridging conformations between multilayer membranes^42^. At this stage, however, the full calculation of this bridging/looping model is probably only feasible through numerical simulations.

It must also be mentioned that in the case of the (beid, short DNA) system, the bottom phases appear to be slightly less well ordered than for all other cases. Indeed, the corresponding SAXS patterns exhibit ill-defined peaks and a *q*^−2.2^ dependence (instead of *q*^−2^) (Fig. 8). This may be tentatively assigned to the fact that salmon DNA may contain slightly more impurities than the other DNA used in the present study. Finally, we note that the (beid, short DNA) phase diagram displays a triphasic region at high clay and DNA concentrations where a nematic and two isotropic phases coexist. Although we did not investigate more precisely the structures and compositions of these phases because of the small phase volumes, these observations are somewhat reminiscent of the phase diagram of gibbsite plates and silica spheres^28^.

## Conclusion

Adding DNA to clay colloidal dispersions essentially gives rise to phase separation and therefore widens the I/N coexistence region. SAXS studies demonstrated that in the clay-rich bottom birefringent phase, the average nanosheet-nanosheet distance between nanosheets is smaller than observed in the absence of DNA, suggesting the onset of a new, DNA-mediated, attractive interaction between the clay particles. We note that similar effects were not previously observed in other mixtures of rods and plates, which suggests a specific interaction between DNA and clay nanosheets. Indeed, a spectroscopic study showed that even though both species bear global negative charges, the DNA adsorbs to some extent on the clay sheets. By analogy with previous studies of the interaction of nucleotides with clay minerals, we infer that the DNA probably adsorb on the nanosheet rims. Long DNA molecules are likely to adopt bridging conformations by adsorbing on several clay nanosheets, thereby effectively suppressing the swollen clay state and pulling the nanosheets together to separations much smaller than they would have in the absence of DNA. Short DNA molecules also adsorb on the nanosheet rims and therefore can bridge clay sheets, with typical separation comparable to the DNA persistence length. Moreover, DNA adsorption increases the negative charge of the clay particles, which, together with an increase in ionic strength, might give rise to Ise-Sogami type attractive electrostatic effects. Our study clearly reveals that the length of DNA molecules and/or the relative size of DNA and clay minerals modify the interaction between these two components. This may bear some significance in soil systems as, up to now, the length and nature of DNA molecules that are known to adsorb on soil components (mainly clay minerals) where they can be protected from degradation has not been explicitly considered.

At this stage, more experiments are clearly required to validate our assumptions about the mechanisms giving rise to attractive interactions in the system. For example, x-ray spectro/microscopy could be used to try to image the location of DNA with respect to the clay nanosheets. Using less polydisperse DNA and varying its contour length could help understanding conformation and steric effects. On the other hand, exploring systematically the influence of the ionic strength and the addition of multivalent cations may also give information about the nature and relevance of electrostatic interactions in such complex systems.

## Electronic supplementary material

Supplementary Information
