# Supplementary material for: Swelling Inhibition of Liquid Crystalline Colloidal Montmorillonite and Beidellite Clays by DNA
Source: Sci Rep. 2018 Mar 12;8:4367. doi: 10.1038/s41598-018-22386-7 (PMC5847546; doi:10.1038/s41598-018-22386-7)
Supplement: Supplementary file 1 — Supplementary Information [file 41598_2018_22386_MOESM1_ESM.docx]

**Supplementary Information**

**Swelling Inhibition of Liquid Crystalline Colloidal Montmorillonite and Beidellite Clays by DNA**

Naoya Yamguchi^1^, Shinya Anraku^1^, Erwan Paineau^2^, Cyrus R. Safinya^3^, Patrick Davidson^2^, Laurent J. Michot^4^, Nobuyoshi Miyamoto^1^

*1 Fukuoka Institute of Technology, Department of Life, Environment and Materials Science, 3-30-1 Wajirohigashi, Higashiku, Fukuoka 811-0295, Japan*

*2 Laboratoire de Physique des Solides, CNRS, Univ. Paris-Sud, Université Paris-Saclay, 91405 Orsay Cedex, France*

*3 Physics Department, Materials Department, and Molecular, Cellular and Developmental Biology Department, University of California, Santa Barbara, California 93106, United States*

*4 Laboratoire Phenix, CNRS−Sorbonne Université−UPMC, UMR 8234, 4, Place Jussieu, 75252, Paris Cedex 5, France*


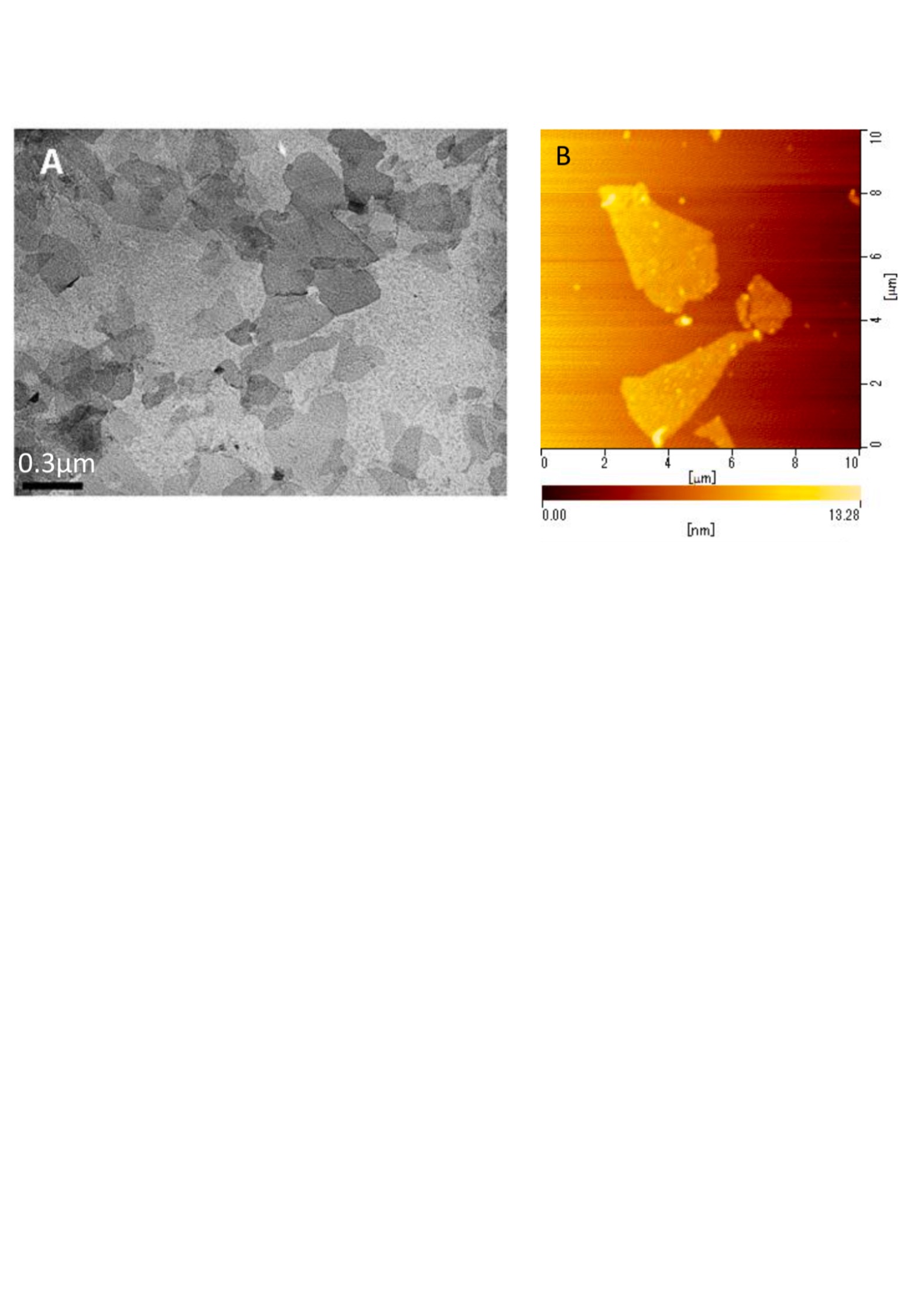


Figure SI1: Typical images by: A) TEM of beidellite clay and B) AFM of montmorillonite clay.


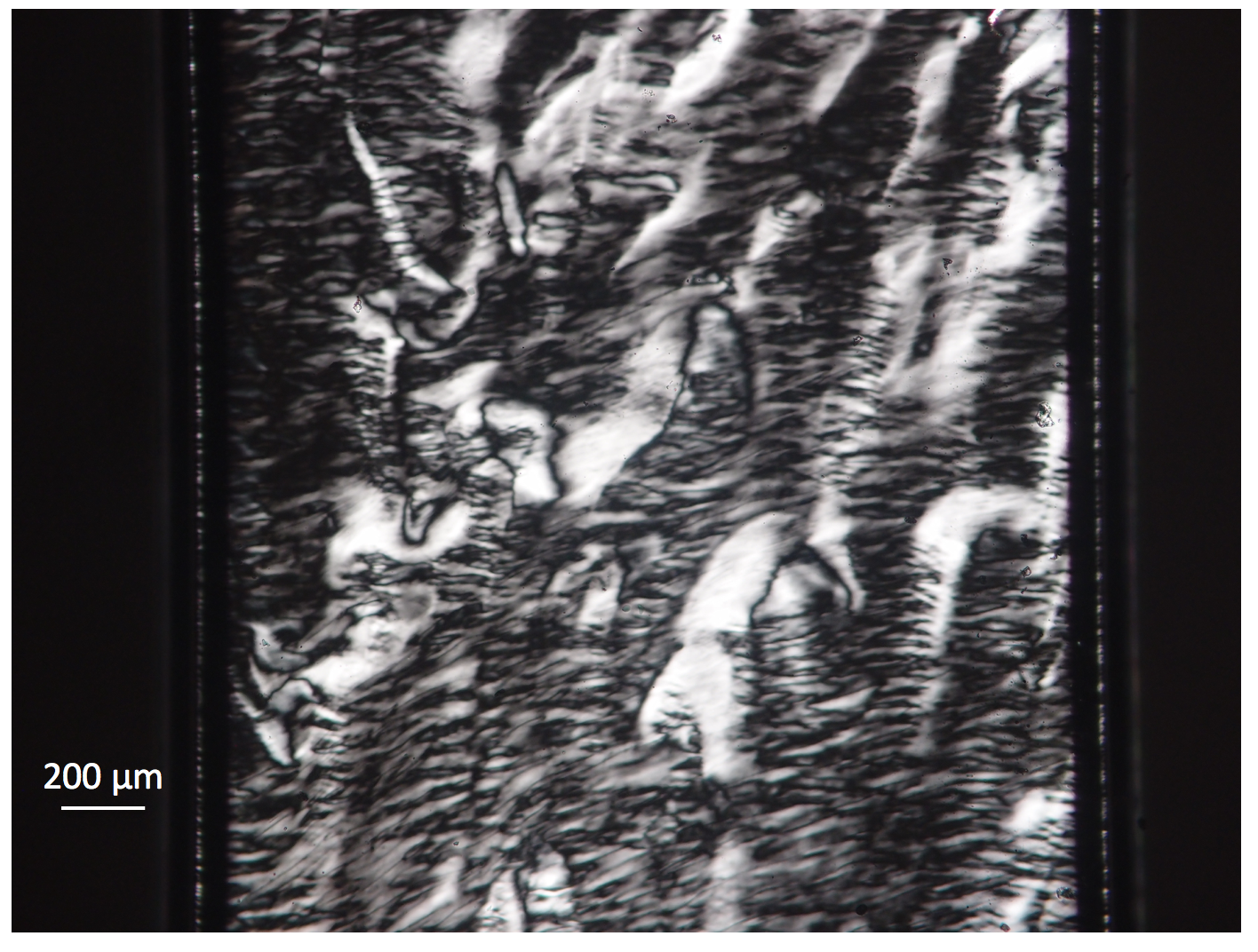


Figure SI2: Nematic texture photograph in polarized light microscopy (polarizer and analyser directions parallel to the edges of the photograph) of a sample of beidellite clay mixed with a purified long DNA (3 kbp).

**Osmotic and depletion pressure calculations**

The osmotic pressure exerted by a solution of DNA particles, if considered as ideal, is given by van’t Hoff law: P = n_b_k_B_T where n_b_ is the bulk number density of DNA particles. The important point here is that n_b_ is actually very small because the DNA molar mass is large. Even in the most favorable case of the “short DNA” of 200 bp and at a typical DNA content of 1 g.L^-1^, with an average molar mass of 650 g mol^-1^ per base pair and neglecting DNA adsorption, the numerical density of DNA rods in the upper phase is only n_b_ = 4.6×10^21^ molecules per m^3^. This leads to an osmotic pressure of 20 Pa, which is much smaller than the ≈ 500 Pa measured in these systems at similar concentrations (see reference 1) that reports our previous determination of the osmotic pressure). Moreover, previous measurements of the osmotic pressure of solutions of short Na^+^-DNA similar to ours, at 1 g L^-1^, are well below 100 Pa (see Figure1 of Reference 2). For the “long DNA” at1 g L^-1^, the osmotic pressure would be even lower as the numerical density is two orders of magnitude smaller. Therefore, it seems that the osmotic pressure exerted by the DNA in the upper phase cannot explain our data. The fact that the clay swelling is restricted by DNA addition therefore strongly suggests that another strong attractive interaction, related to DNA, must come into play.

To discount the depletion interactions, we made a rough calculation of their order of magnitude. For this purpose, we neglect the influence of electrostatic effects and we only consider the interaction between two clay platelets. (see reference 3) The osmotic pressure due to depletion by the DNA short rods, between two nanosheets almost at contact, is n_b_k_B_T where n_b_ is the bulk number density of DNA particles, k_B_ is the Boltzmann constant and T the temperature. Even in the most favorable case of the “short DNA” of 200 bp and at a typical DNA content of 1 g.L^-1^, with an average molar mass of 650 g.mol^-1^ per base pair and neglecting DNA adsorption, the numerical density of DNA rods in the phase is only n_b_ = 4.6×10^21^ molecules per m^3^. This leads to an osmotic pressure of 20 Pa, which is much smaller than the ≈ 500 Pa measured in these systems at similar concentrations. For the long DNAs, the osmotic pressure is even one or two orders of magnitude lower (depending on which one).

**References**

1) E. Paineau, e. a. Liquid-Crystalline Nematic Phase in Aqueous Suspensions of a Disk-Shaped Natural Beidellite Clay. J Phys Chem B 113, 15858-15869 (2009).

2) E. Raspaud et al, Do Free DNA Counterions Control the Osmotic Pressure?, Physical Review Letters, **84**, 2533 (2000)

3) Lekkerkerker, H. N. W. & Tuinier, R. *Colloids and the Depletion Interaction*. (Springer, 2011), page 89.”
